# Effects of erythropoietin on osteoblast in the tooth extraction socket in mice periodontitis model

**DOI:** 10.3389/fphys.2022.987625

**Published:** 2022-10-06

**Authors:** Ju-Eun Bae, Sung-Min Hwang, Yam Prasad Aryal, Tae-Young Kim, Wern-Joo Sohn, Seo-Young An, Ji-Youn Kim, Chang-Hyeon An, Youngkyun Lee, Yong-Gun Kim, Jin-Woo Park, Jae-Mok Lee, Jae-Young Kim, Jo-Young Suh

**Affiliations:** ^1^ Department of Periodontology, School of Dentistry, IHBR Kyungpook National University, Daegu, South Korea; ^2^ Department of Biochemistry, School of Dentistry, IHBR Kyungpook National University, Daegu, South Korea; ^3^ Pre-Major of Cosmetics and Pharmaceutics, Daegu Haany University, Gyeongsan, South Korea; ^4^ Department of Oral and Maxillofacial Radiology, School of Dentistry, IHBR Kyungpook National University, Daegu, South Korea; ^5^ Department of Dental Hygiene, College of Health Science, Gachon University, Incheon, South Korea

**Keywords:** bone formation, erythropoietin, human periodontal ligament fibroblast cell, local delivery, MC3T3-E1 cells, osteoblast, periodontitis, tooth loss

## Abstract

Periodontitis is an excessive inflammatory event in tooth-supporting tissues and can cause tooth loss. We used erythropoietin (EPO), which has been reported to play an important role in bone healing and modulation of angiogenesis, as a therapeutic agent *in vivo* and *in vitro* experimental models to analyze its effect on periodontitis. First*,* EPO was applied to *in vitro* MC3T3-E1 cells and human periodontal ligament fibroblast (hPDLF) cells to examine its function in altered cellular events and gene expression patterns. *In vitro* cultivation of MC3T3-E1 and hPDLF cells with 10 IU/ml EPO at 24 and 48 h showed an obvious increase in cell proliferation. Interestingly, EPO treatment altered the expression of osteogenesis-related molecules, including alkaline phosphatase (ALP), bone morphogenetic protein-2 (BMP-2), and osteocalcin (OC) in MC3T3-E1 cells but not in hPDLF cells. In particular, MC3T3-E1 cells showed increased expression of ALP, BMP-2, and OC on day 5, while hPDLF cells showed increased expression of BMP-2, and OC on day 14. Based on the *in vitro* examination, we evaluated the effect of EPO on bone formation using an experimentally-induced animal periodontitis model. After the induction of periodontitis in the maxillary left second M, 10 IU/ml of EPO was locally applied to the extraction tooth sockets. Histomorphological examination using Masson’s trichrome (MTC) staining showed facilitated bone formation in the EPO-treated groups after 14 days. Similarly, stronger positive reactions against vascular endothelial growth factor (VEGF), cluster of differentiation 31 (CD31), runt-related transcription factor 2 (RUNX2), and osteocalcin (OC) were detected in the EPO-treated group compared to the control. Meanwhile, myeloperoxidase, an inflammatory marker, was decreased in the EPO-treated group on days 1 and 5. Overall, EPO facilitates bone healing and regeneration through altered signaling regulation and modulation of inflammation in the osteoblast cell lineage and to a lesser extent in hPDLF cells.

## Introduction

Periodontitis is inflammation of the tooth-supporting tissues due to Gram-negative bacteria, and results in periodontal pocket formation, gingival recession, and gradual destruction of the periodontal ligament and alveolar bone (Struillou et al., 2010). Several studies have shown that in periodontal lesions, lipopolysaccharides (LPS) synthesized by Gram-negative bacteria induce the production of excessive reactive oxygen species (ROS)-like substances and increase oxidative stress (Maruyama et al., 2011; Kim et al., 2020a). Severe forms of periodontitis result in tooth loss, which can affect the patient’s quality of life (Cochran, 2008; Struillou et al., 2010). Replacement of lost teeth with dental implants in periodontally compromised patients is a therapeutic goal in dental science. However, traditional periodontal therapies are only effective for relieving inflammation but not for replacing missing teeth (Chen et al., 2010). Implant placement at the correct location with sufficient bone mass is required for comfortable eating and overall satisfaction. Moreover, bone tissue characteristics, such as bone quality and density, are important factors for proper attachment of dental implants (Ribeiro-Rotta et al., 2011).

Bone formation and remodeling activities are essential (Amler et al., 1960) for the differentiation and maturation of remaining cells following tooth loss (Shiozawa et al., 2010). After tooth loss, vascular cells, immune cells, osteoblasts, and remaining periodontal ligament fibroblasts (PDLF) are present in the tooth root extraction socket (Cardaropoli et al., 2003). Among the remaining cell population, osteoblasts differentiate from mesenchymal stem cells (MSCs) (Shiozawa et al., 2010) and are involved in the deposition of bone matrix (Caetano-Lopes et al., 2007) and the regulation of osteoclastic and metabolic activities (Guo et al., 2014). PDLF cells are reported to have similar characteristics to MSCs in humans and can be observed between 1 and 7 days after extraction in animal model experiments (Cardaropoli et al., 2003; Seo et al., 2004; Wang et al., 2018). It has been suggested that human PDLF (hPDLF) cells have regenerative capacity and affect osteogenesis in the extraction socket (Devlin and Sloan, 2002; Fujii et al., 2010; Sato and Takaoka, 2015; Horibe et al., 2021). In addition, osteoblasts and PDLF in the tooth extraction socket mainly act in periodontal tissue regeneration, including the PDL and alveolar bone (Tuna et al., 2015). In addition, migration and differentiation of osteoblasts and angiogenic cells are known to play critical roles in tissue regeneration after tooth extraction (Kanyama et al., 2003).

Erythropoietin (EPO), a 34 kDa glycoprotein hormone and a member of the hematopoietic class I cytokine superfamily (Lacombe and Mayeux, 1999), is a growth hormone that stimulates neovascularization and has been used to treat several types of anemia (Hiram-Bab et al., 2015). EPO is involved in the proliferation and differentiation of hematopoietic stem cells (HSCs) and hematopoietic progenitor cells (HPCs) (Wang et al., 2018). Furthermore, the non-hematopoietic effects of EPO and its ability to promote bone formation and tissue protection by preventing ischemia and inflammatory responses has also been studied (Mcgee et al., 2012). EPO promotes the differentiation of MSCs into osteoblasts, and is involved in the interactions between osteoblasts and osteoclasts to increase bone formation (Shiozawa et al., 2010; Sun et al., 2012). EPO has also been shown to modulate excessive inflammation by antagonizing and modulating pro-inflammatory cytokines such as tumor necrosis factor-alpha (TNF-α) (Erbayraktar et al., 2003; Rolfing, 2014). Moreover, EPO has been reported to accelerate the rate of recovery and new bone formation by increasing the number of blood vessels, fibers, and osteoblasts in a mouse mandibular distraction osteogenesis model (Mihmanli et al., 2009). EPO is also known to induce MSCs to increase new bone formation in rats and humans (Sun et al., 2012; Li et al., 2015; Wang et al., 2018), while endogenous EPO is known to play a role in balancing osteogenic and adipogenic differentiation of BMSCs (Suresh et al., 2019).

Given that EPO affects bone healing and angiogenesis, we examined the precise cellular effects of EPO *in vitro* using hPDLF and MC3T3-E1 cells, as well as its bone-healing capacity *in vivo* in tooth extraction sockets of a ligature-induced periodontitis mouse model.

## Materials and methods

### Animals

All experiments were conducted according to the guidelines of the Intramural Animal Use and Care Committee of Kyungpook National University, School of Dentistry (KNU 2015–136). The adult C57BL/6 mice were housed in a room temperature of 22 ± 2°C, humidity of 55% ± 5%, and artificial illumination with lights on from 05:00 to 17:00 h. Food and water were provided *ad libitum*.

### *In vitro* cell culture: hPDLF and MC3T3-E1

The hPDLF were harvested from the extracted premolars of three patients at the Department of Periodontology, Kyungpook National University Dental Hospital. Their ages ranged from 10 to 20 years, and the teeth were extracted for orthodontic treatment. This study was performed in accordance with the Institutional Review Board of Kyungpook National University Hospital (KNUH-74005-745), and informed consent was obtained from all donors. The hPDLF cells were isolated and cultured as previously described (Min et al., 2020). All hPDLF cells between 4th and 7th passage were used in the present study.

MC3T3-E1 cells, which were isolated from the calvaria of C57BL/6 mice, were obtained from American Type Culture Collection (CRL-2593; Manassas, United States). MC3T3-E1 cells were cultured in α-minimum essential medium (α-MEM; cat. No. SH30265.01; Hyclone, United States) supplemented with 10% FBS, 100 U/ml penicillin, and 100 μg/ml streptomycin. The cells were incubated at 37°C in a humidified atmosphere containing 5% CO_2_. The culture medium was refreshed every 2 days.

### Cell proliferation assay

The concentration-dependent effects of recombinant human erythropoietin (EPO; cat. No. PHC9634, Gibco™, Life Technologies, United States) on the proliferation of hPDLF and MC3T3-E1 cells was determined using a Cell Counting Kit-8 (CCK-8; cat. No. CK04-11, Dojindo, Japan) according to the manufacturer’s instructions. The hPDLF cells were seeded at a density of 5 × 10^3^ cells/well in 96-well plates in low-glucose DMEM supplemented with 10% FBS. The MC3T3-E1 cells were seeded at a density of 5 × 10^3^ cells/well in 96-well plates in α-MEM supplemented with 10% FBS. After 24 h, the medium was changed to low-glucose DMEM or α-MEM supplemented with 2% FBS and various concentrations of EPO (0, 2.5, 5, 10, 20, and 50 IU/ml). After 24 and 48 h of EPO application, 10 μl of CCK-8 solution was added to each well, followed by incubation for 45 min at 37°C in a humidified atmosphere containing 5% CO_2_. The absorbance was measured using a microtiter plate reader (Fluostar OPTIMA, BMG Labtech, Germany) at 450 nm. Cell proliferation was expressed as a percentage of untreated control cells. All experiments were conducted independently in triplicate.

### Real-time polymerase chain reaction (PCR) analysis

The hPDLF cells were seeded at a density of 1 × 10^5^ cells/well in a 6-well plate in low-glucose DMEM supplemented with 10% FBS. MC3T3-E1 cells were seeded at a density of 1 × 10^5^ cells/well in 6-well plates in α-MEM supplemented with 10% FBS. After 24 h, the medium was replaced with low-glucose DMEM or α-MEM supplemented with 2% FBS and 50 μg/ml ascorbic acid (cat. No. A4403; Sigma-Aldrich, St. Louis, MO, United States), 10 mM beta-glycerophosphate (cat. No. G9422; Sigma-Aldrich, United States), and 100 nM dexamethasone (cat. No. D4902; Sigma-Aldrich, United States) with 0 or 10 IU/ml erythropoietin (EPO). On days 1, 3, 5, 7, and 14, total RNA was isolated from the cultured cells using TRIzol reagent (cat. No. 15596026, Invitrogen™, United States). cDNA was generated from 2 µg of total RNA using the iScript™ cDNA Synthesis Kit (cat. No. 1708881, Bio-Rad, United States). Real-time PCR was performed to quantify gene expression with iQ SYBR Green Supermix (cat. No. 1708890, Bio-Rad, United States). The genes examined in this study, including alkaline phosphatase (ALP), bone morphogenetic protein-2 (BMP-2), osteocalcin (OC), and a housekeeping gene (glyceraldehyde-3 phosphate dehydrogenase, GADPH), are listed in Table 1. Each sample was analyzed in triplicate. The results of the assays were normalized to GAPDH levels. Real-time PCR results were analyzed using the 2^-△△C^_T_ method (Livak and Schmittgen, 2001).

**TABLE 1:** List of primers used for RT-PCR analysis of hPDLF cells and MC3T3-E1 cells F: Forward, R: Reverse, ALP: Alkaline phosphatase, OC: Osteocalcin, BMP-2: Bone morphogenetic protein-2, GADPH: Glyceraldehyde-3 phosphate dehydrogenase.

Genes	Primer sequences for hPDLF cells	Primer sequences for MC3T3-E1 cells
ALP	F: 5‘- CTG​CCA​TCC​TGT​ATG​GCA​ATG -3’	F: 5′-ATC​TTT​GGT​CTG​GCT​CCC​ATG-3′
	R: 5‘- AGA​CTG​CGC​CTG​TAG​TTG​TTG -3’	R: 5′-TTT​CCC​GTT​CAC​CGT​CCA​C-3′
BMP-2	F: 5‘- TTT​GGA​CAC​CAG​GTT​GGT​GAA -3’	F: 5′-CCA​CAG​CCT​TCA​TGT​CCA​AG-3′
	R: 5‘- ACG​AAT​CCA​TGG​TTG​GCG​T -3’	R: 5′-GGC​AGA​GAG​AGA​GGA​CAG​GG-3′
OC	F: 5‘- TAG​TGA​AGA​GAC​CCA​GGC​GCT​A -3’	F: 5′-CCG​TCA​TTC​CGG​ATT​ACA​TGA​G-3′
	R: 5‘- TCA​CAG​TCC​GGA​TTG​AGC​TCA -3’	R: 5′-TCA​CTG​GTC​CCT​GGG​ATG​TCC-3′
GAPDH	F: 5‘- CCGCATCTTCTTTTGCGT -3’	F: 5′-GGT​GCT​GAG​TAT​GTC​GTG​GA-3′
	R: 5‘- AGT​TAA​AAG​CAG​CCC​TGG​TG -3’	R: 5′-CAG​TTG​GTG​GTG​CAG​GAT​G-3′

### *In vivo* tooth extraction model

Six-week-old C57BL/6 male mice were used in the experiments (30 mice in total). The mice were randomly divided into two groups: vehicle control (15 mice per group) and experimental group (15 mice per group). In both groups, the maxillary left second M were ligated for 5 days with 5-0 black silk (cat. No. SK54510, AILEE Co., Ltd., Korea) to induce periodontitis under anesthesia, as previously described (Adhikari et al., 2019). The maxillary right second M of each mouse were left untreated as controls. The maxillary left second M were extracted using an explorer under anesthesia after 5 days of tie. In the control group, 2 μl of Pluronic^®^ F-127 (CAS:9003-11-6, Sigma-Aldrich, Germany), which is a nontoxic and nonionic amphiphilic surfactant and, in aqueous solutions (hereafter PF127Aq), undergoes a reversible thermogelling process, similar to other Pluronics (Jalaal et al., 2017), and 0.01% dimethyl sulfoxide (DMSO; CAS:67-68-5, Duchefa Biochemie, Netherlands) were applied to the extraction socket, while the experimental group was treated with 2 μl of a mixture comprising EPO, Pluronic^®^ F-127, and 0.01% DMSO. The final EPO concentration in the mixture was maintained at 10 IU/ml. A gas-tight Hamilton syringe (Lot No. 541628, Hamilton company, Reno, United States) was used to infuse 2 μl of the mixture directly into the socket, according to a previous study (Adhikari et al., 2019). The wound site was sealed using fibrin sealant (Tisseel; cat. No. 1501261, Baxter Healthcare, United States). The mice were sacrificed 1, 5, and 14 days after the application of EPO in the socket.

### Histology and immunostainings

For histological analysis, the excised mouse maxillary bones were fixed with 4% paraformaldehyde in phosphate-buffered saline (PBS) and maintained overnight at 4°C. After rinsing twice with PBS, the specimens were decalcified using ethylenediaminetetraacetic acid (EDTA; Cat No. GMB 002, Dongin Biotech, Korea) solution for 4 weeks at 4°C. The EDTA solution was changed every 2 days. The fixed specimens were dehydrated with ethanol and xylene and embedded in paraffin. Sections (5 μm) were cut and mounted on slides coated with poly-l-lysine (Muto Pure Chemicals, Japan). After fixation and decalcification, the harvested maxillae were dissected parallel to the palatal rugae, and both the right and left maxillary second M were observed on the same slide to identify the location of the extraction socket, as previously described (Adhikari et al., 2021). For histological examination, sections were stained with hematoxylin and eosin and Masson’s trichrome (MTC) to detect connective tissue, newly formed bone, and collagen fibers in the socket healing stage (Suvik and Effendy, 2012).

Immunostaining was performed to observe changes in the structure of the socket, as previously described (Adhikari et al., 2019). Primary antibodies against vascular endothelial growth factor (VEGF; cat. No. bs-0279R; 1 × 400; Bioss Antibodies, United States), and cluster of differentiation 31 (CD31; cat. No. ab28364; 1:100, Abcam, United Kingdom) were used to detect the blood vessel distribution. Primary antibodies against inflammatory marker myeloperoxidase (MPO; cat. No. bs-4943R; 1:250; Bioss Antibodies, United States), osteocalcin (cat. ab93876; 1:250, Abcam, United Kingdom), and runt-related transcription factor 2 (RUNX2; cat. No. ab192256; 1:1000; Abcam, United Kingdom) were used to investigate bone formation and differentiation. Biotinylated anti-rabbit or anti-rat IgG was used as the secondary antibody, and a diaminobenzidine tetrahydrochloride (DAB) reagent kit (cat. No. C09-12; GBI Labs, United States) was used to detect binding of the primary antibody to the sections.

### Image analysis

All slides stained for histology and immunohistochemistry were acquired using a DM2500 microscope (Leica Microsystems, Wetzlar, Germany) equipped with a DFC310 FX digital CCD camera (Leica Microsystems). ImageJ software was used for the statistical analysis of level of bone formation on the designated regions of MTC staining tissues as described previously (Adhikari et al., 2019). Briefly, the original MTC stained images were converted to RGB images and they were deconvolved by ImageJ using color deconvolution plugin. When a constant threshold was set, the area and integrated density of collagen fibres in the region of interest were estimated. Four different images of 50 μm^2^ from 5 different samples from randomly selected specimens were used. For the quantitative analysis of RUNX2 and OC, positive cells of them were counted from 3 images of 100 μm^2^ (positive for immunostaining) selected randomly from 5 different specimens.

### Statistical analysis

All experiments were performed in triplicate. SPSS software (SPSS 24.0 KO; SPSS Inc.) was used to perform statistical analyses. The examined data were assessed using analysis of variance (ANOVA) followed by Tukey’s post hoc test. All values are expressed as the mean ± standard error (SE). *p*-values < 0.05 were considered statistically significant.

## Results

### Proliferation of hPDLF and MC3T3-E1 cells

**FIGURE 1:**
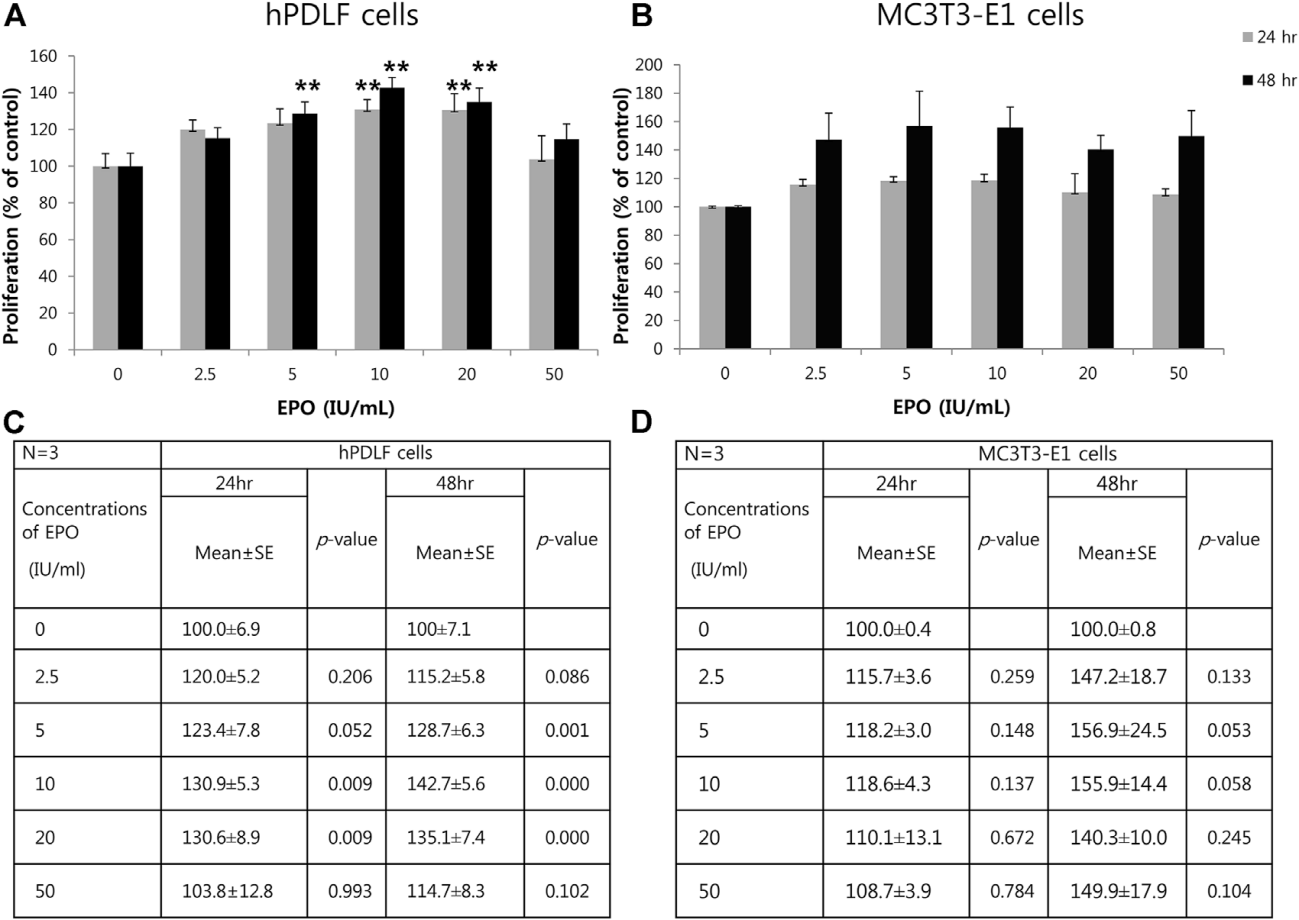
Effect of EPO on proliferation of hPDLF cells **(A,C)** and MC3T3-E1 cells **(B,D)**. After 24 and 48 h incubation, a cell proliferation assay is conducted using a Cell Counting Kit-8 (CCK-8). The experiments are conducted three times independently. Treatment groups with 5, 10, and 20 IU/ml EPO show significantly higher values than the control group (*p* < 0.01) at 24 and 48 h in hPDLF cells **(A,C)**. In MC3T3-E1 cells **(B,D)**, higher proliferation rates are reported for EPO-treated groups, which show the highest values at 5 and 10 IU/ml EPO at both 24 and 48 h, than the control group. Data are shown as mean ± standard error (SE). *Statistically significant differences compared to the untreated control (*p* < 0.05). **Statistically significant differences compared to the untreated control (*p* < 0.01).

To examine the effects of EPO on the proliferation of hPDLF and MC3T3-E1 cells, various concentrations of EPO (0, 2.5, 5, 10, 20, and 50 IU/ml) were employed, and proliferation assays were conducted using the CCK-8 kit after 24 and 48 h (Figure 1). The proliferation of hPDLF cells showed a significant increase (*p* < 0.01) as the EPO concentration increased to 20 IU/ml, and then decreased at 50 IU/ml (*p* > 0.05) (Figure 1A). The application of 10 and 20 IU/ml EPO resulted in the highest level of cell proliferation among all concentrations tested. The values were approximately 1.3–1.4-fold higher than untreated control group at 24 h and 48 h, respectively (Figure 1A). The proliferation rate of MC3T3-E1 cells showed an increasing trend at all concentrations, but with no statistical significance (*p* > 0.05) (Figure 1B). The applications of 5 and 10 IU/ml EPO resulted in the highest cellular proliferation among all concentrations tested (Figure 1B) and the values were approximately 1.18- and 1.56-fold higher than those of the untreated control group at 24 h and 48 h, respectively (Figure 1B).

### Expression levels of BMP-2 and osteoblast differentiation markers

**FIGURE 2:**
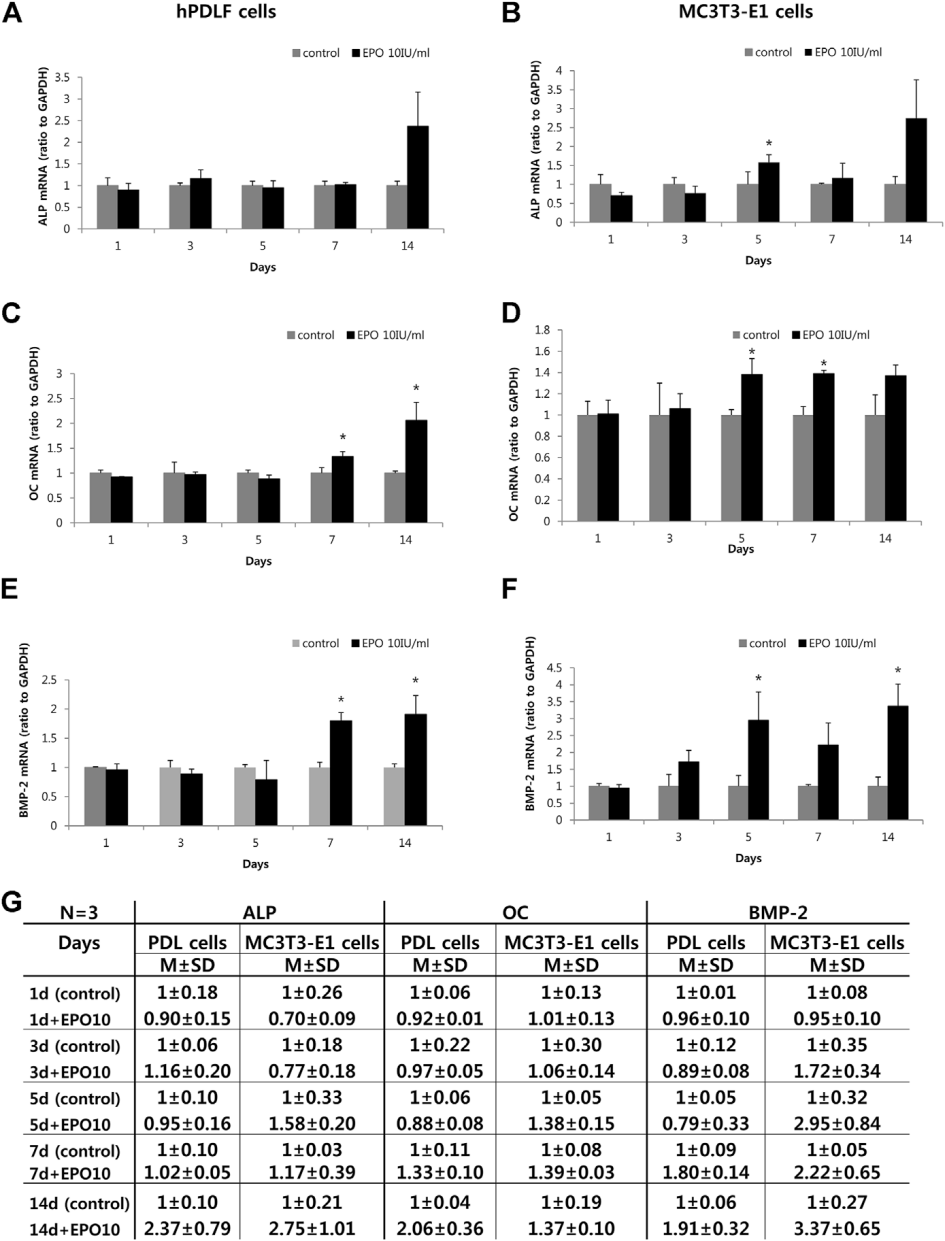
Effect of EPO on osteogenic gene markers of hPDLF cells **(A,C,E,G)** and MC3T3-E1 cells **(B,D,F,G)**. Treatment with 10 IU/ml EPO show the highest alkaline phosphatase (ALP) expression on day 14, but no significant effect is observed on the hPDLF cells **(A)**. In contrast, MC3T3-E1 cells show significantly higher ALP expression on day 5 **(B)**. Significantly higher expression level of osteocalcin (OC) is observed on day 7 and 14 on the hPDLF cells **(C)**, but the MC3T3-E1 cells show significantly higher values on days 5 and 7 **(D)**. Significantly higher expression level of bone morphogenetic protein-2 (BMP-2) is observed on day 7 and 14 in hPDLF cells **(E)**, but appear more rapidly, on days 5 and 14, in the MC3T3-E1 cells **(F)**. The experiments are conducted three times independently. Data are shown as mean (M) ± standard deviation (SD). *Statistically significant differences compared to the untreated control (*p* < 0.05).

The potential role of EPO in osteogenic function was observed by examining the expression levels of ALP, OC and BMP-2 in hPDLF and MC3T3-E1 cells (Figure 2A-G) by real-time PCR. Specifically, the expression levels of BMP-2 and osteoblast differentiation markers (ALP and OC) were examined at different time intervals after treatment with EPO. In the hPDLF cells, the expression of ALP, an early stage osteoblast differentiation marker (Ortiz-Vigón et al., 2017) was upregulated in the treatment group only on day 14 (Figure 2A). In particular, the expression of OC, a late-stage osteoblast differentiation marker (Li et al., 2017), was significantly higher on days 7 and 14 in the EPO-treated group (*p* < 0.05) (Figure 2C). Similarly, the expression levels of BMP-2, which are known to promote bone and vascular remodeling (Cai et al., 2012; Chen et al., 2012), were significantly higher in the EPO-treated group on days 7 and 14 (*p* < 0.05) (Figure 2E). In the MC3T3-E1 cells, the EPO-treated groups showed significantly higher ALP expression on day 5 (*p* < 0.05); however, the expression was not significantly higher on day 14 (Figure 2B). The expression level of OC was also higher on days 5 and 7 (*p* < 0.05) (Figure 2D), and the expression of BMP-2 was significantly upregulated on days 5 and 14 in the EPO-treated group (*p* < 0.05) (Figure 2F).

### Histomorphology of bone healing socket after EPO treatment

**FIGURE 3:**
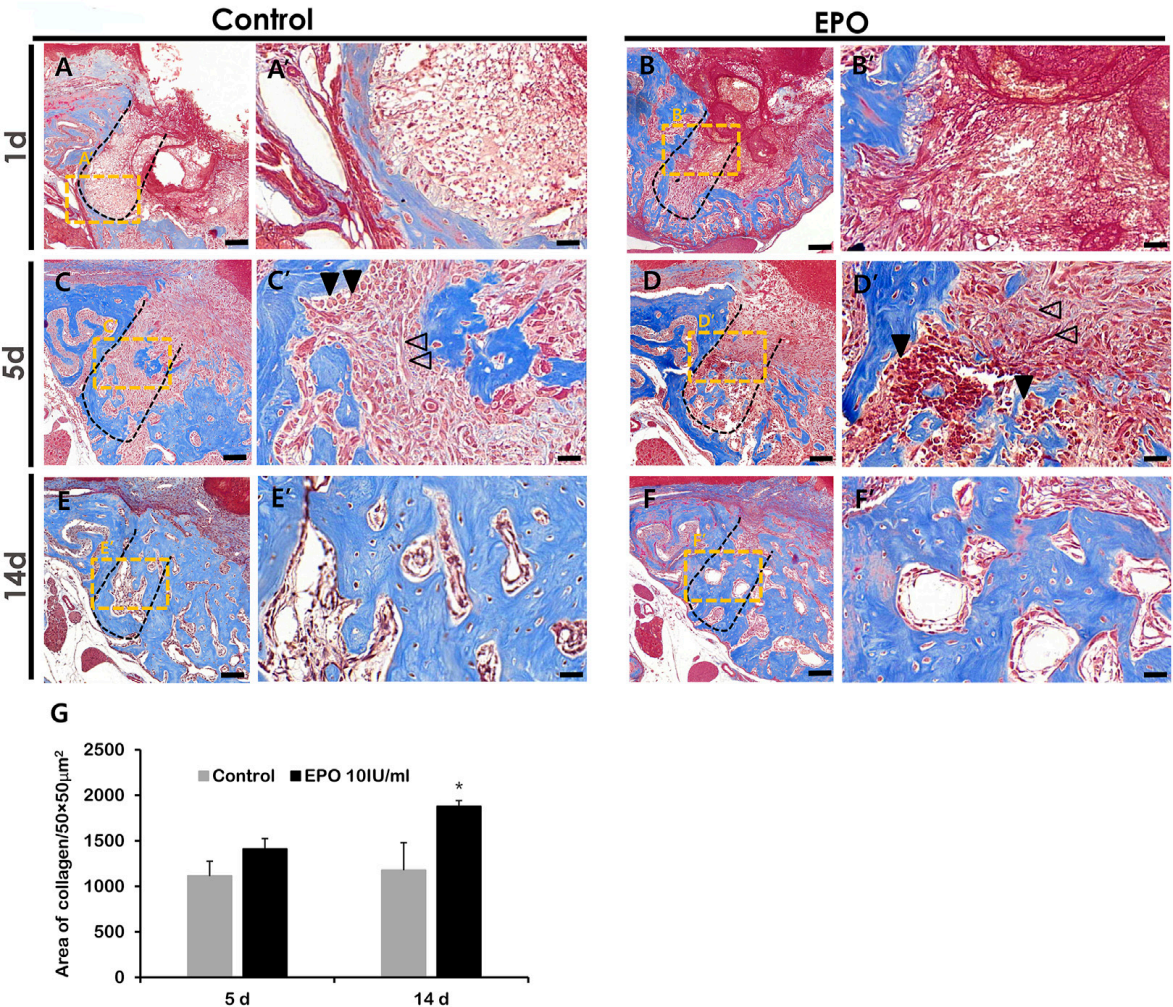
Representative photomicrographs of alveolar tissue sections stained with Masson’s trichrome (MTC) in the control group **(A,C,E)** and EPO group **(B,D,F)** at 1, 5 and 14 days. On day 1, residual hPDLF is observed in the socket walls in both groups, with more vascular structures visible in the experimental group **(A′,B′)**. On day 5, early signs of new bone formation are more obvious in the experimental group compared to the control group **(C′,D′)**. On day 14, both groups show mature bone **(E′,F′)**, but the experimental group show more newly formed bone in the tooth socket **(E′,F′)**. The volume of newly synthesized collagen (blue) is less in the control group **(C,E)** than in EPO group **(D,F)**. Statistical analysis for newly synthesized collagen in the tooth socket **(G)**. Black arrow heads point to osteoblasts, and transparent arrows to fibroblast-like cells. Scale bar: X = 100 μm, X’ = 20 μm (X′ is a higher magnification of X). *Statistically significant differences compared to the untreated control (*p* < 0.05).

The histology of the healing extraction socket in the mouse periodontitis model was examined by MTC staining (Figure 3), as described previously (Adhikari et al., 2019). On day 1 post-treatment, residual PDLF was observed in both groups, and the coagulum filled the sockets (Figures 3A,B). In addition, more vascular structures were observed in the EPO group (Figure 3A′,B′). On day 5, histological alterations in new bone formation were observed along the margin and at the bottom of the socket in both groups (Figures 3C,D). Compared to the control, spindle-shaped fibroblast-like cells were observed in the experimental group (Figure 3C′,D′). Similarly, an increased number of blood vessel-like cells and developing bone tissues with dense collagen formation (blue) were observed in the experimental group (Figure 3C′,D′,G). On day 14, gradual calcification of bony tissues was observed in both groups (Figures 3E,F); however, in the EPO-treated group, a slight increase in bone formation was observed in the tooth root socket compared to the control (Figure 3E′,F′,G).

### Altered immunostaining patterns of proteins after EPO treatment

**FIGURE 4:**
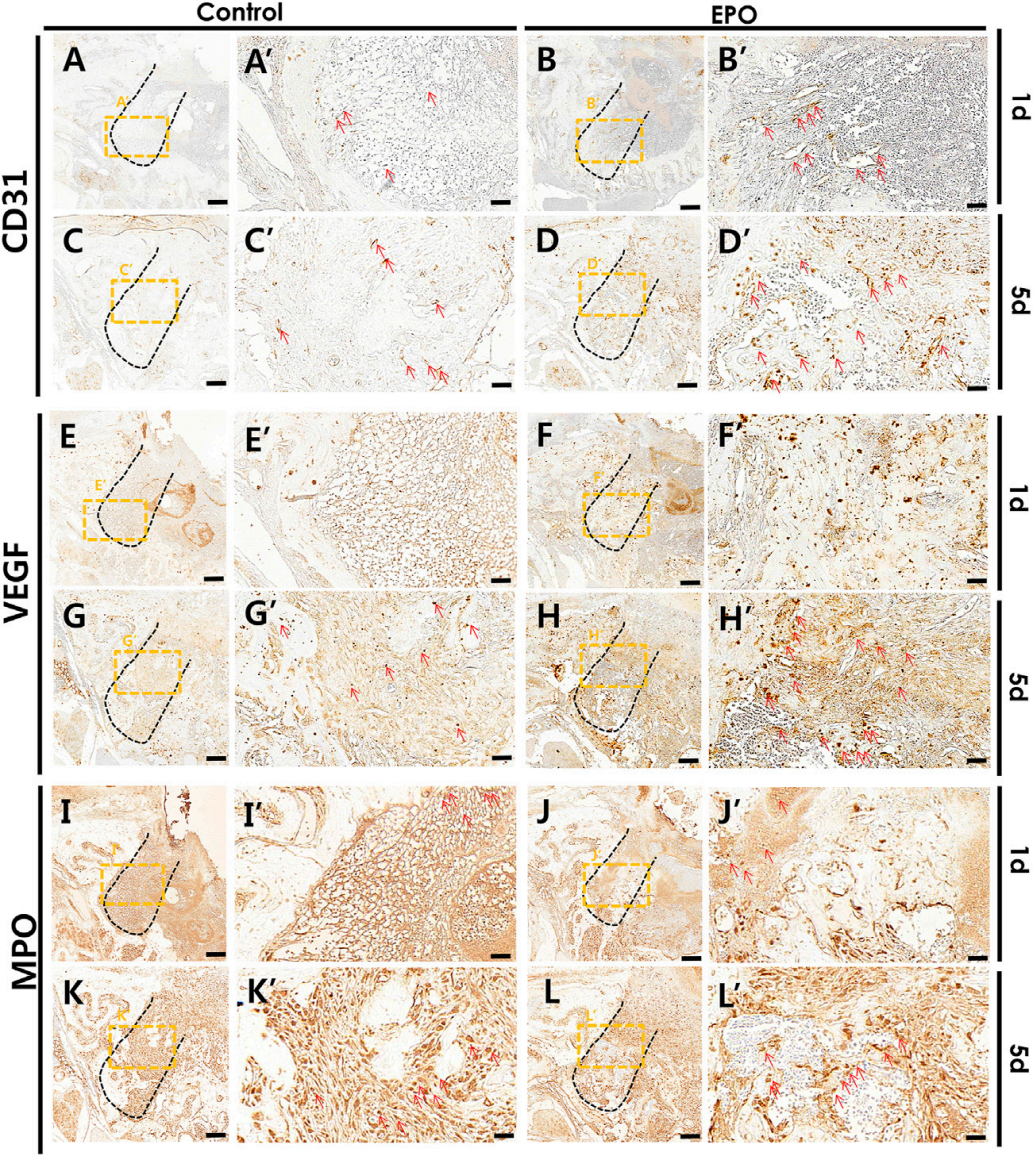
Representative photomicrographs of alveolar tissue sections stained with vascular endothelial markers and the inflammatory marker in the control group **(A,C,E,G,I,K)** and EPO group **(B,D,F,H,J,L)** at 1 and 5 days. On day 1, the EPO group show stronger expression of cluster of differentiation 31 (CD31) and vascular endothelial growth factor (VEGF) in the entire extraction socket, especially in the apical and middle one-third of the regions compared to the control **(A′,B′,E′,F′)**. On day 5, the localization patterns are intensified in the EPO group compared to those observed on day 1 **(B′,D′,F′,H′)**. The EPO group also show a much stronger localization pattern, especially in the coronal 1/3 compared to that in the apical 1/3 and middle 1/3 regions **(B′,D′,F′,H′)**. In contrast, the EPO group showed decreased immunolocalization of myeloperoxidase (MPO) in both 1- and 5-day specimens **(I′,J′,K′,L′)**. Red arrows point to positive cells against CD31 **(A–D)**, VEGF **(E–H)**, and MPO **(I–L)**. Scale bar: X = 100 μm, X’ = 20 μm (X′ is higher magnification of X).

**FIGURE 5:**
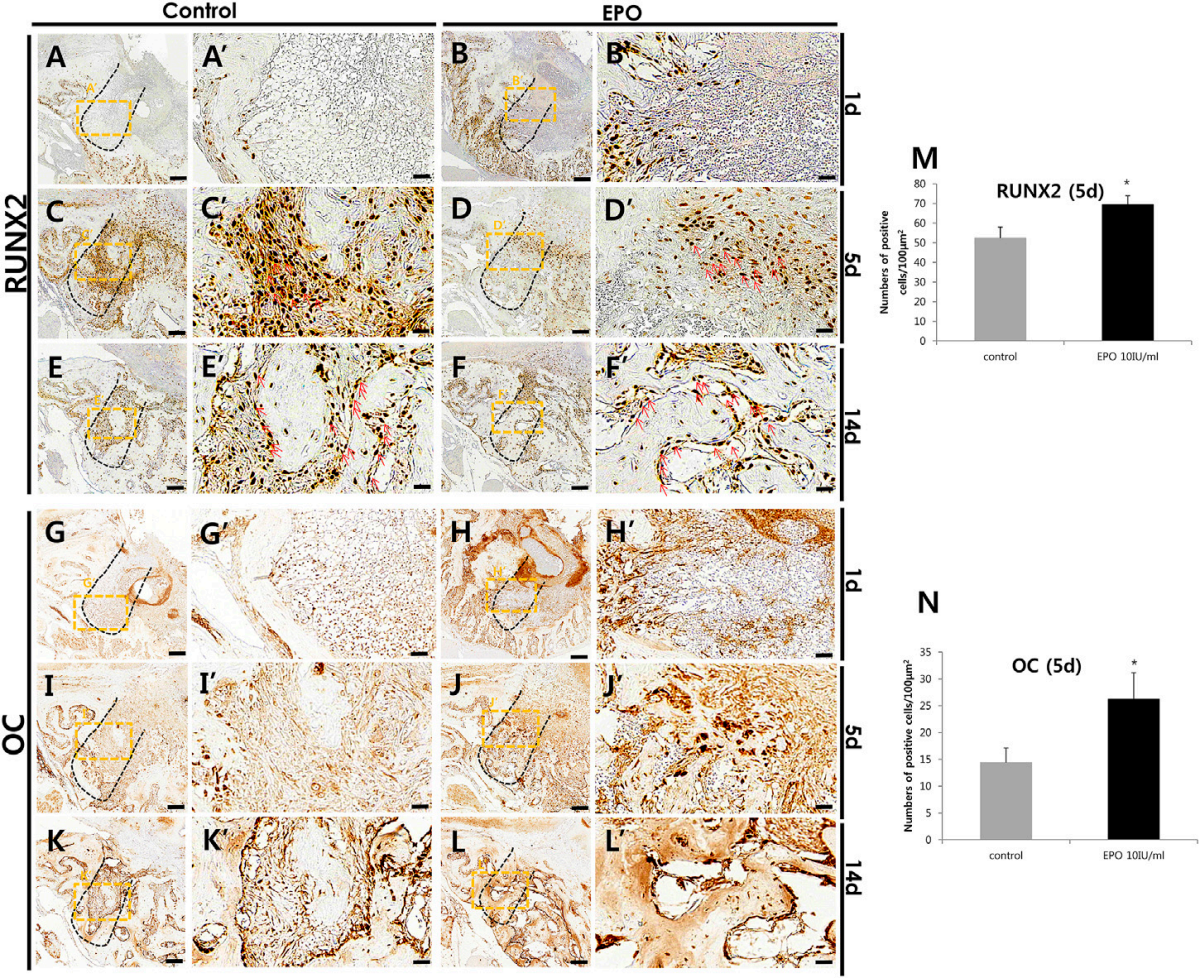
Representative photomicrographs of alveolar tissue sections stained with osteoblast markers in the control group **(A,C,E,G,I,K)** and EPO group **(B,D,F,H,J,L)** at 1, 5, and 14 days. Compared to control, the localization of Runt-Related Transcription Factor 2 (RUNX2) in the EPO group is more intense on day 1; however, its localization patterns are decreased on day 5 **(A′,B′,C′,D′)**. In 1-day specimens, the expression of RUNX2 is also more visible along the socket wall and in the apical 1/3 region of the experimental group **(A′,B′)**. In contrast, osteocalcin (OC) localization on the day 1 specimen is weaker in the EPO group; however, its localization pattern is increased on day 5 **(G′,H′,I′,J′)**. Specifically, OC-positive cells are observed only along the socket wall in the control group, but in the entire socket in the EPO group **(I′,J′)**. However, on day 14, RUNX2 and OC are strongly expressed in the entire socket of both groups **(E′,F′,K′,L′)**. Red arrows point to positive cells against RUNX2 **(C′,D′,E′,F′)**. Histological analysis using RUNX2 **(M)** and OC **(N)** at 5 days. Scale bar: X = 100 μm, X’ = 20 μm (X′ is higher magnification of X). *Statistically significant differences compared to the untreated control (*p* < 0.05).

Immunostaining of blood vessel (CD31 and VEGF), inflammatory (MPO) and osteogenic (RUNX2 and OC) markers was performed to define the precise roles of EPO during healing of the extraction socket and bone regeneration. On day 1 and day 5, an obvious increase in the staining of CD31 and VEGF, were detected in the EPO-treated groups (Figures 4A–H). Increased positive cells against CD31 and VEGF mainly localized in middle part of the tooth root socket of the EPO-treated group when compared with the control group. In contrast, the anti-MPO immunostained area was comparatively decreased in the EPO-treated group at 1 day (Figure 4I′,J′). On day 5, the anti-MPO immunostained area was similar or less in the EPO-treated group compared to that in the control group (Figure 4K′,L′). In contrast, stronger immunostaining of the pre-osteoblastic marker RUNX2 in the EPO-treated group was observed on day 1 (Figure 5A′,B′). As shown by MTC staining (Figure 3C′,D′,G), EPO-treated specimens showed increased newly synthesized collagen bundles with RUNX2 positive cells compared to the control on day 5 (Figure 5C′,D′,M). On day 14, as was examined in histological observations (Figure 3), newly formed bone matrices were occupied in both control and experimental groups (Figures 5E,F). On days 1 and 5, EPO-treated specimens showed an increased number of OC-positive cells compared to the control (Figure 5G′,H′,I′,J′,N). In addition, on day 14, more anti-OC immunostained area was observed in the entire socket on EPO-treated specimens compared to the control (Figure 5K′,L′). The numbers of RUNX2 and OC positive cells in the root socket was counted and prepared for statistical analysis (Figure 5M,N). On day 5, the number of positive cells against RUNX2 and OC was significantly increased compared to the control (*p* < 0.05) (Figure 5M,N). The positive cells against RUNX2 were detected on the surface margin of bone matrices, usually osteoblast cells are examined (Figure 5E′,F′). EPO-treated group showed the denser and increased immunostained area on the surface of bony tissue (Figure 5F′).

## Discussion

EPO has been reported to promote bone formation by inducing MSCs and secretion of bone morphogenetic proteins (BMP-2 and −6) from HSCs and HPCs (Rolfing et al., 2014b). However, few studies have examined the regenerative roles and bone-healing capacity of EPO in the dental field. Therefore, we aimed to investigate the role of EPO in bone formation in dental-derived cell populations, including osteoblasts, hPDLF cells, and tooth extraction sockets, through *in vitro* and *in vivo* studies, for which we have already established experimental protocols in previous reports (Adhikari et al., 2019; Kim et al., 2020b). Numerous cell populations can be observed in the tooth root socket following dental extraction; these include PDLF, blood vessel-associated pericytes, immune cells, and osteoblasts, all of which are involved in the process of bone healing (Devlin and Sloan, 2002; Cardaropoli et al., 2003). Osteoblasts and hPDLF cells are known to play important roles in bone formation through cellular differentiation processes (Tomokiyo et al., 2008; Hwang and Horton, 2019; Kim et al., 2021). As these cells contribute to bone formation in the extraction socket, we sought to identify and examine the cellular effects of EPO on MC3T3-E1 and hPDLF cells *in vitro* (Figures 1, 2). Our *in vitro* results showed that EPO modulated the cellular physiology of these cells in a time- and concentration-dependent manner (Figure 1). Cellular proliferation and differentiation are important events in wound healing and tissue regeneration (Dhivya et al., 2018). In our study, hPDLF and MC3T3-E1 cell proliferation assays after EPO treatment revealed a dose-dependent effect of EPO on cell proliferation, as previously reported (Mcgee et al., 2012). Similar observations of growth patterns with 10 IU/ml of EPO have also been reported for the proliferation of hPDLF cells, human osteoblastic cells (Guo et al., 2014; Zheng et al., 2019), and mesenchymal stromal cells (Rolfing et al., 2014a). Interestingly, EPO treatment increased and facilitated the expression levels of ALP, OC, and BMP-2 in MC3T3-E1 cells when compared to those in hPDLF cells (Figure 2).

ALP, OC, and BMP-2 play vital roles in osteogenesis (Aubin et al., 1995; Herzog et al., 2014; Lee et al., 2017). In this study, MC3T3-E1 cells treated with EPO showed increased expression of ALP, an early osteoblast differentiation marker (Lee et al., 2017), similar to previous findings (Shiozawa et al., 2010; Li et al., 2014; Wang et al., 2018). Similarly, EPO facilitates the significantly higher expression of BMP2, which plays a major role during osteoblast differentiation and the organization of vascular networks (Riley et al., 1996; Herzog et al., 2014), in the osteoblast cell line MC3T3-E1 rather than in PDLF (Park et al., 2006; Shiozawa et al., 2010; Nair et al., 2013). Based on these results and those of previous reports, we speculate that EPO modulates BMP2 signaling to facilitate bone formation (Holt et al., 2005; Shiozawa et al., 2010; Granholm et al., 2013).

To confirm these *in vitro* results in an *in vivo* condition, we evaluated the effects of EPO on bone formation using an *in vivo* mouse periodontitis model by examining the histomorphology and immunostaining (Figures 3–5).

Modulation of inflammation is considered essential for the bone healing process (Martins-Junior et al., 2016; Aoyagi et al., 2018). Neutrophils are recruited immediately after tooth extraction and produce activated MPO, an inflammatory marker, by phagocytosis (Aoyagi et al., 2018). Excessive or low amounts of neutrophils in tissues disrupt tissue homeostasis and lead to periodontitis (Magan-Fernandez et al., 2020). Periodontitis is an inflammatory condition, and tissue regeneration is slower in cases of tooth extraction in the presence of periodontitis than in healthy tooth extraction (Kim et al., 2020b; Min et al., 2020; Adhikari et al., 2021). Decreased MPO immunostaining in the EPO-treated group would suggest that EPO might involve in modulation of early inflammation as were examined in a range of diseases (Figure 4; Lee et al., 2007; Mateus et al., 2017; Zhou et al., 2017; Kim et al., 2020b; Min et al., 2020; Adhikari et al., 2021). However it is necessary to further examine the immunomodulation roles of EPO in the periodontitis in near future. As previously reported, EPO is a multifunctional cytokine with anti-inflammatory properties and is involved in regulating the regenerative capacity of bone formation from periodontitis (Silva et al., 2021).

Blood vessel formation is an important process that facilitates bone formation by delivering oxygen, hormones, growth factors, and osteoblasts (Shiozawa et al., 2010; Wu et al., 2014). In accordance with previous reports, EPO induces and facilitates vascular tissue formation Figure 4; (Erbayraktar et al., 2003; Rolfing, 2014; Hiram-Bab et al., 2015); and plays roles in bone formation Figure 5; (Amler, 1969; Cardaropoli et al., 2003). Therefore, the blood supply facilitated by EPO treatment is thought to be important for early wound healing and bone formation (Sivaraj and Adams, 2016; An et al., 2017). As examined by MTC staining, CD31, and VEGF localization, increased bone formation and newly synthesized collagen bundles near blood vessel-forming regions were observed in the middle of the tooth root socket in the EPO-treated group on day 5 (Figure 3C′,D′, Figure 4C′,D′,G′,H′). These results are similar to those of previous reports, in which EPO played a role in bone fracture healing and angiogenesis (Garcia et al., 2011; Bakhshi et al., 2013).

Moreover, the stronger localization pattern of pre-osteoblastic marker, RUNX2 on day 1 in experimental group suggest that EPO would facilitate the osteogenesis (Figure 5). OC is secreted by osteoblasts and plays a major role in bone mineralization or homeostasis of calcium ions (Aubin et al., 1995). Our results suggest that EPO has a modulating during the proliferation and differentiation of osteoblasts during healing and regeneration of bone, as reported previously (Shiozawa et al., 2010; Wang et al., 2018).

On day 5, prior to bone formation, we observed positive reactions against RUNX2 and OC in the extraction socket. The histology of the tooth socket at 5 and 14 days after treatment revealed that EPO facilitated collagen synthesis and mineralized tissue formation, with increased localization patterns of RUNX2 and OC in the experimental group, as previously reported (Figures 3, 5; Mcgee et al., 2012; Li et al., 2015). These results suggest that EPO plays a critical role in bone formation by modulating osteoblast differentiation.

In this study, EPO was shown to increase the early healing rate of the extraction socket in mice with ligature-induced periodontitis. EPO may maintain the integrity of hPDLF cells, improve the proliferation of osteoblasts in the extraction socket and promote bone formation through inflammation control and angiogenesis. Importantly, MC3T3-E1 cells responded more sensitively to EPO; although hPDLF cells are also affected by EPO, it is thought that a period of differentiation is required owing to their stem cell-like properties (Tomokiyo et al., 2018). This study is meaningful in that EPO can shorten the time taken for bone regeneration and promote the possibility of bone quality improvement for implant placement under conditions of periodontitis clinically, in the same way that it is used to treat fractures.

## Data Availability

The original contributions presented in the study are included in the article/Supplementary Material, further inquiries can be directed to the corresponding authors.
